# Patterns of Treatment Switching in Multiple Sclerosis Therapies in US Patients Active on Social Media: Application of Social Media Content Analysis to Health Outcomes Research

**DOI:** 10.2196/jmir.5409

**Published:** 2016-03-17

**Authors:** Valéry Risson, Deepanshu Saini, Ian Bonzani, Alice Huisman, Melvin Olson

**Affiliations:** ^1^ Novartis Pharma AG Basel Switzerland; ^2^ IMS Health Haryana India; ^3^ IMS Health London United Kingdom

**Keywords:** Internet, multiple sclerosis, outcomes assessment, drug switching

## Abstract

**Background:**

Social media analysis has rarely been applied to the study of specific questions in outcomes research.

**Objective:**

The aim was to test the applicability of social media analysis to outcomes research using automated listening combined with filtering and analysis of data by specialists. After validation, the process was applied to the study of patterns of treatment switching in multiple sclerosis (MS).

**Methods:**

A comprehensive listening and analysis process was developed that blended automated listening with filtering and analysis of data by life sciences-qualified analysts and physicians. The population was patients with MS from the United States. Data sources were Facebook, Twitter, blogs, and online forums. Sources were searched for mention of specific oral, injectable, and intravenous (IV) infusion treatments. The representativeness of the social media population was validated by comparison with community survey data and with data from three large US administrative claims databases: MarketScan, PharMetrics Plus, and Department of Defense.

**Results:**

A total of 10,260 data points were sampled for manual review: 3025 from Twitter, 3771 from Facebook, 2773 from Internet forums, and 691 from blogs. The demographics of the social media population were similar to those reported from community surveys and claims databases. Mean age was 39 (SD 11) years and 14.56% (326/2239) of the population was older than 50 years. Women, patients aged 30 to 49 years, and those diagnosed for more than 10 years were represented by more data points than other patients were. Women also accounted for a large majority (82.6%, 819/991) of reported switches. Two-fifths of switching patients had lived with their disease for more than 10 years since diagnosis. Most reported switches (55.05%, 927/1684) were from injectable to oral drugs with switches from IV therapies to orals the second largest switch (15.38%, 259/1684). Switches to oral drugs accounted for more than 80% (927/1114) of the switches away from injectable therapies. Four reasons accounted for more than 90% of all switches: severe side effects, lack of efficacy, physicians’ advice, and greater ease of use. Side effects were the main reason for switches to oral or to injectable therapies and search for greater efficacy was the most important factor in switches to IV therapies. Cost of medication was the reason for switching in less than 0.5% of patients.

**Conclusions:**

Social intelligence can be applied to outcomes research with power to analyze MS patients’ personal experiences of treatments and to chart the most common reasons for switching between therapies.

## Introduction

The Internet is rapidly becoming the first source of general and specific information on illnesses and treatments for a large number of people worldwide. Surveys have reported that between 70% and 75% of online users in the United States and Europe search for health care information [1-4]. Social media platforms are commonly used by patients seeking to connect with others with the same disease and as a source of medical information. In the United Kingdom, Facebook is reported as the fourth most popular source of health information [5]. In the United States, 42% of respondents to a recent survey said that they had used social media to find out about a health care issue, 25% had discussed a health-related experience, and 20% had joined a health community or forum [6].

For researchers, this explosion of online activity has generated a treasure trove of digital data that can be mined for insights into various aspects of health-seeking behavior and treatments. In the areas of infectious disease and public health, social network messages have been analyzed (eg, syndromic surveillance, disease sentiment analysis, or studies of drug abuse epidemiology using social media) [7-9]. Social media are a well-established source of patient-reported information on pharmacovigilance. Beyond media managed by pharmaceutical companies, microblogs such as Twitter have been used as a source of information about adverse events from a variety of medications [10] or to assay opinions about treatments [11].

In recognition of this value of the Internet and digital media, the European Medicines Agency in its guideline on good pharmacovigilance practices recommends that marketing authorization holders should regularly screen Internet or digital media (Web sites, Web pages, blogs, vlogs, social networks, Internet forums, chat rooms, health portals) under their management or responsibility for potential reports of suspected adverse reactions [12].

Despite this intense activity, social media analysis has rarely been applied to the study of specific questions in outcomes research. To test the validity of social media information and the applicability of social intelligence to outcomes research, we developed a comprehensive process that blends automated listening with the filtering and analysis of data by life sciences-qualified analysts and physicians. The process was designed to combine the advantages of purely manual and purely automated analyses. Manual coding in content analysis studies is superior to automatic content analysis in capturing complex semantic relationships between concepts or recognizing irony or sarcasm, which are frequently used in the informal conversation typical of social media. Automated analysis systems are superior in their sheer computing power, required to scan large datasets for relevant lexical entities, coding, and statistical analysis.

The mix of manual and automatic content analysis has been applied in other settings [9], but to our knowledge the applicability of the method to social media and health outcomes research has not been evaluated.

In this pilot study, we validated our social intelligence process by analyzing the representativeness of the social media population with multiple sclerosis (MS). To apply the analysis to outcomes research, we then used the collected data to study patterns of treatment switching to and from oral therapies in the MS population.

## Methods

Social intelligence analysis combines automated listening to social media conversations with the filtering and analysis of data by human experts. As such, it is a noninterventional, retrospective database analysis of data available in the public domain.

The population was patients with MS from the United States. Patients who did not mention their location country or mentioned their location as not in the United States were excluded from the switchover analysis. Data sources for user-generated content were Facebook, Twitter, blogs, and online forums. The sites were selected because they are highly active, with a large number of users, and the content is publicly available and not redacted. Only publicly available social media conversations were included in the listening process and no password-restricted information was accessed. No patient-identifiable information available on social media was collected. Age, sex, and geographic location of social media users were collected when available.

### Social Intelligence Process

**Figure 1:**
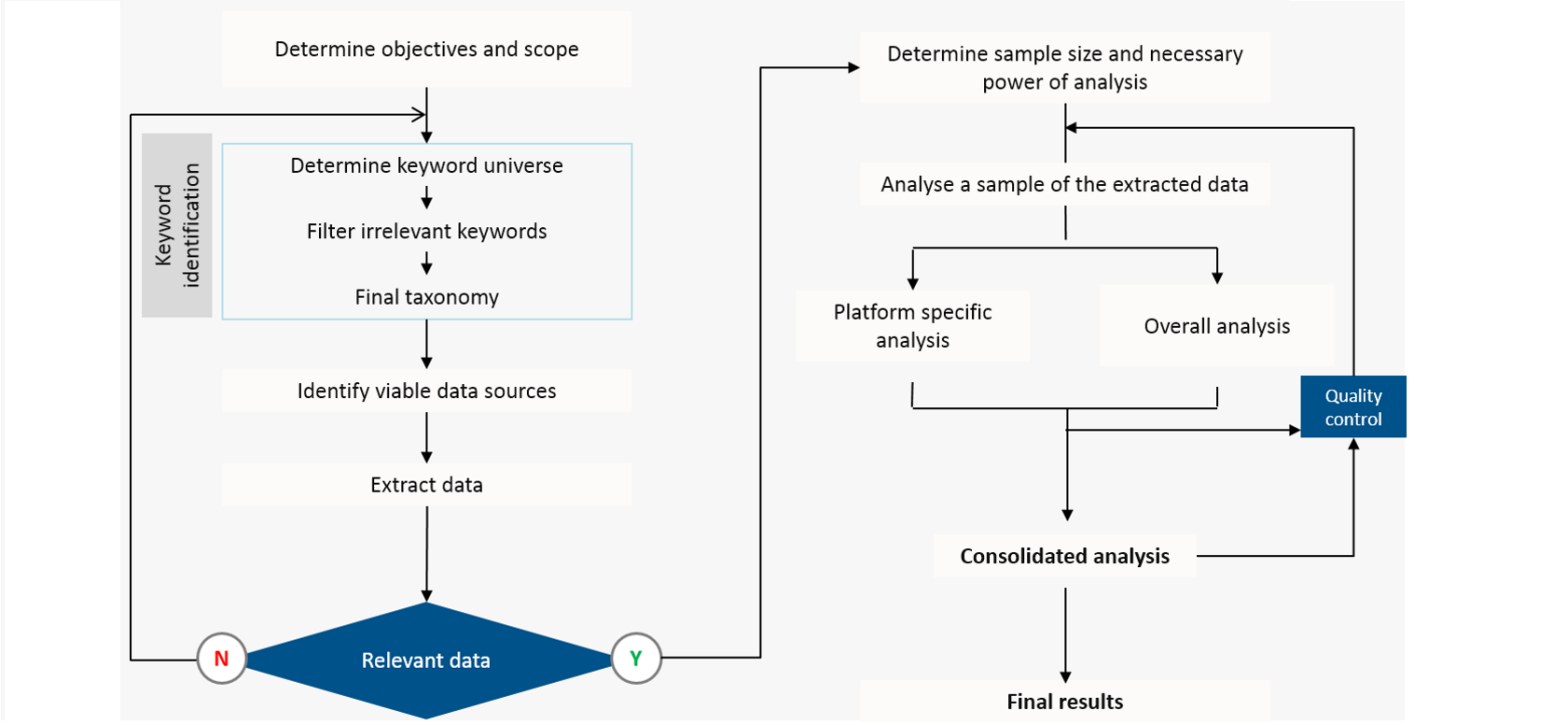
Flowchart of the social intelligence analysis process. The system was trained to identify switchovers through a set of keyword patterns such as “switched from [brand A] to [brand B],” “moving from [brand A] to [brand B],” “stopping [brand A] starting [brand B],” and similar expressions.

The overall process is illustrated in Figure 1. Data were collected from social media by monitoring the sources automatically and extracting semantic information. The system identified lexical entities in conversations related to the objectives of switching patterns between oral and injectable therapies for MS. Switchovers were identified through a set of keyword patterns, such as “switched from [brand A] to [brand B],” “moving from [brand A] to [brand B],” “stopping [brand A] starting [brand B],” and similar expressions. As proxies for oral therapies, the trade names Tecfidera (dimethyl fumarate), Gilenya (fingolimod), and Aubagio (teriflunomide) were used as terms. Proxies for subcutaneously and intramuscularly injectable therapies were the common BRACE therapies: Betaseron (interferon beta-1b), Rebif (interferon beta-1a), Avonex (interferon beta-1a), Copaxone (Glatiramer acetate), and Extavia (interferon beta-1b). In addition, Tysabri (natalizumab) and Novantrone (mitoxantrone) were included as representatives of intravenous (IV) infusion treatments. Search terms also included common misspellings of brand names.

The identification of potential switches was performed by the automatic system based on a set of predefined categories: potential switches; category of conversations as information sharing or information seeking; themes of conversation as cost, efficacy, side effects, or adherence; and conversation sentiment as positive or negative. For potential switches, the system was trained to recognize conversations containing switching patterns, terms, brands involved in a switch, and the potential reason for the switch. The data were filtered for relevance to the study objectives. The identification and grouping of content was done by the automated system. The automatic identification was followed by manual analysis of the complete set of records to eliminate errors and record the involved brands and reasons for switchovers.

The objectives of the analysis were (1) to explore the feasibility of using social media analysis to address outcomes questions in health care that focus on a population of MS patients; (2) to validate the representativeness of the social media population by comparing the characteristics of the database population with data on MS patients obtained by other methods, such as medical records, patient advocacy groups, or general practitioners; and (3) to use the validated database to analyze switching patterns and reasons for switching between oral and injectable classes of MS therapies.

This study was designed, implemented, and reported in accordance with the Guidelines for Good Pharmacoepidemiology Practices of the International Society for Pharmacoepidemiology [13], the Strengthening the Reporting of Observational Studies in Epidemiology (STROBE) guidelines [14], and with the ethical principles laid down in the Declaration of Helsinki [15]. The secondary data source used for the analysis meets all the US Health Insurance Portability and Accountability Act (HIPAA) compliance standards, ensuring patient anonymity. As such, approval from an Institutional Review Board was not necessary.

### Statistical Analysis

The information extracted by the automated system was organized by social platform (ie, Twitter, Facebook, online discussion forums, and blogs). The data were further stratified monthly for each platform. To generate representative samples for the population dataset for manual review, random samples were extracted for each month and for each platform based on a 95% confidence interval with a 5% margin of error. These data were subjected to manual analysis by life sciences-qualified analysts, who were guided by a physician, to confirm the representativeness of the samples. After the review of these samples, validation sample sets were generated for three random months for each platform using the preceding methodology to cross-validate the findings derived from the sample set.

## Results

Social media conversations were monitored between October 1, 2013 and October 31, 2014. A total of 49 forums and 152 blogs were used for the analysis. All publicly available Facebook pages were searched for the relevant semantic information. The automated system extracted 25,073 social media data points (unique posts on any specific media platform) in this time period. A total of 22,887 relevant data points were identified: 8672 from Twitter, 6919 from Facebook, 6505 from Internet forums, and 791 from blogs. Of these, 10,260 data points were sampled by the analysts for a detailed manual review: 3025 from Twitter, 3771 from Facebook, 2773 from Internet forums, and 691 from blogs.

The demographics of the population are shown in Table 1. Mean age was 39 (SD 11) years and 14.56% (326/2239) of the population was older than 50 years. The mean time since diagnosis was 6.8 (SD 4.5) years, but one-third of the population reported more than 10 years since diagnosis. Women, patients aged between 30 and 49 years, and those diagnosed for more than 10 years were represented by more data points than other patients were. Women also accounted for a large majority (82.6%, 819/991) of reported switches. Mean time since diagnosis in patients who switched medication was 7.5 (SD 4.3) years and two-fifths of switching patients had lived with their disease for more than 10 years since diagnosis.

**Table 1:** Demographics of the analyzed population.

Variable		All patients N=3911	Switching patients n=991
Sex (female), n (%)		3001 (76.73)	819 (82.6)
Age (years), mean (SD)		39 (11)	40 (12)
**Age distribution (years), n (%)**		n=2239	n=515
	<20	11 (0.49)	7 (1.4)
	20-29	442 (19.74)	102 (19.8)
	30-39	602 (26.89)	139 (27.0)
	40-49	858 (38.32)	167 (32.4)
	50-59	210 (9.38)	57 (11.1)
	60-69	113 (5.05)	43 (8.3)
	>70	3 (0.13)	0
Years since diagnosis, mean (SD)		6.8 (4.5)	7.5 (4.3)
**Distribution of time since diagnosis, n (%)**		n=992	n=429
	0-6 months	97 (9.8)	8 (1.9)
	6-12 months	38 (3.8)	7 (1.6)
	1-3 years	198 (20.0)	106 (24.7)
	4-8 years	239 (24.1)	97 (22.6)
	9-10 years	88 (8.9)	38 (8.9)
	>10 years	332 (33.5)	173 (40.3)

As a validation of the representativeness of the social media population, the characteristics were compared with those from a survey in three US communities in Texas, Ohio, and Missouri [16]. As shown in Table 2, the sex ratios and age distributions were numerically similar across all populations. There were minor differences in age distribution between the populations, most notably the greater percentage of patients younger than 30 years in this study population compared with the community survey populations. Overall, the identified social media population corresponded well to those in the communities used as comparator. A further comparison with published data confirmed that the percentage of women in the study population (76.73%, 3001/3911) was numerically similar to published rates from three large US administrative claims databases: MarketScan (76.4%), PharMetrics Plus (76.5%), and Department of Defense (76.4%) [17].

**Table 2:** Comparison of characteristics of the study population with those of MS populations from three US communities [16].

Variable		Study population N=3911	Texas (Lubbock and 19-County surrounding area) N=182	Missouri (Independence and Sugar Creek) N=106	Ohio (Lorain County) N=320
Sex (female)		76.73%	80.1%	81.1%	74.1%
**Age distribution (years)**		N=2239			
	<30	20.23%	10.4%	3.8%	3.1%
	30-39	26.89%	32.4%	12.3%	10.6%
	40-49	38.32%	26.9%	37.7%	30.3%
	50-59	9.38%	9.3%	32.1%	31.9%
	60-69	5.05%	2.2%	10.4%	12.5%
	>70	0.13%	0.5%	3.8%	11.6%

**Figure 2:**
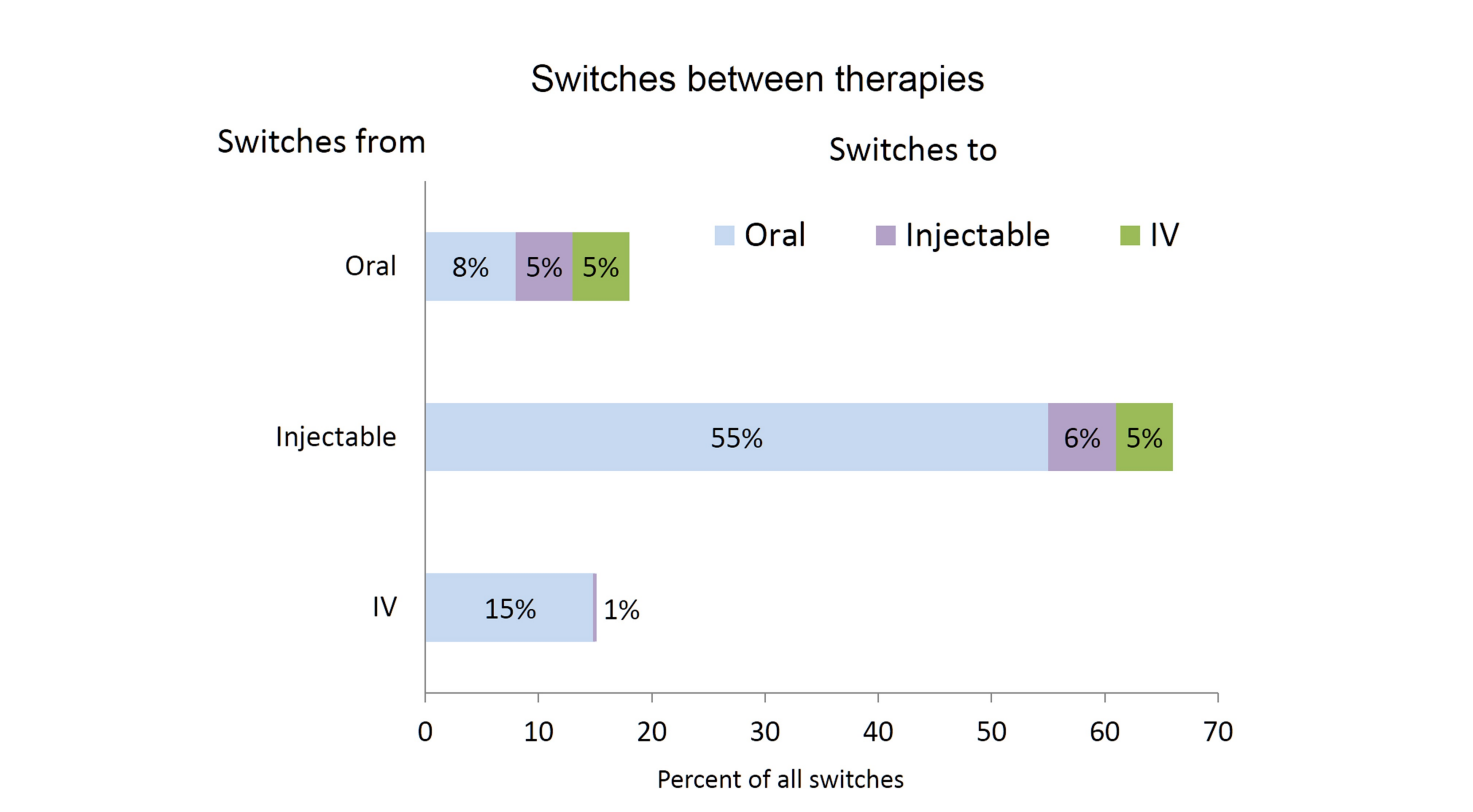
Percentages of all switches representing switches from oral, injectable, and IV therapies, respectively.

A total of 1684 data points were identified as treatment switches. Switches were most frequent among patients on injectable therapies who were represented by 1114 of 1684 (66.15%) data points for switches (Figure 2). The largest proportion of reported switches (55.05%, 927/1684) were from injectable medications to oral drugs. The second largest single switch (15.38%, 259/1684) was from IV therapies to orals. Overall, switches to oral drugs accounted for 78.74% (1326/1684) of all switches. Switches from oral therapies to other drug classes were described in 9.50% (160/1684) of the data points.

**Figure 3:**
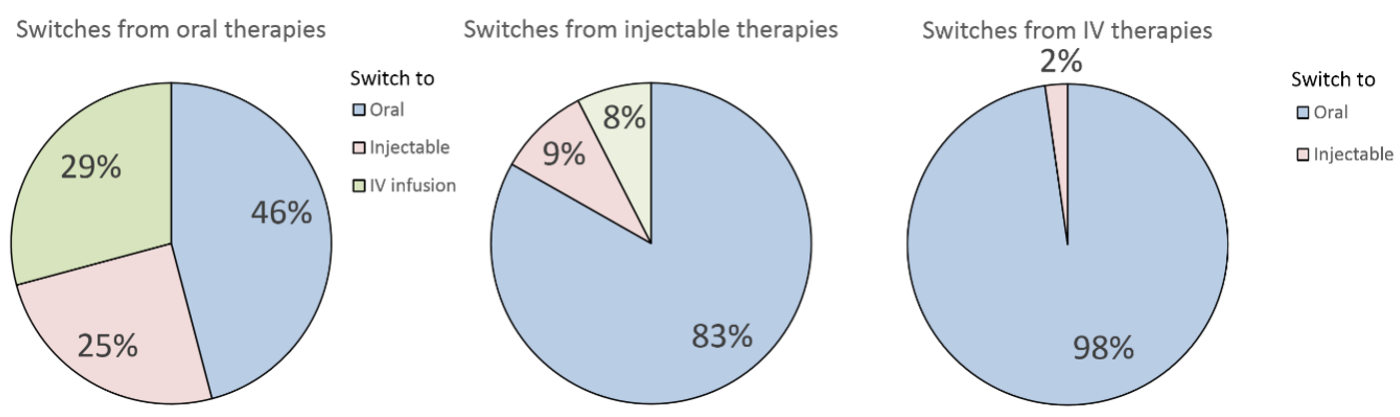
Percentages of switches from oral, injectable, and IV therapies, respectively, to other therapies.

Relative rates of switches away from oral, injectable, and IV therapies, respectively, are shown in Figure 3. The largest percentages of switches in both groups were to oral therapies. Switches to oral drugs accounted for more than 80% (927/1114) of switches away from injectable therapies. There was a less clear trend among switches away from orals. Although in this group, as in the others, the most common single switch was to other oral therapies, such switches accounted for less than half of the total. There seemed to be no clear preference for injectable compared to IV drugs in terms of switches. Switches away from IV therapies were overwhelmingly (97.7%, 259/265) to orals, with a small number of switches to injectable therapies.

**Figure 4:**
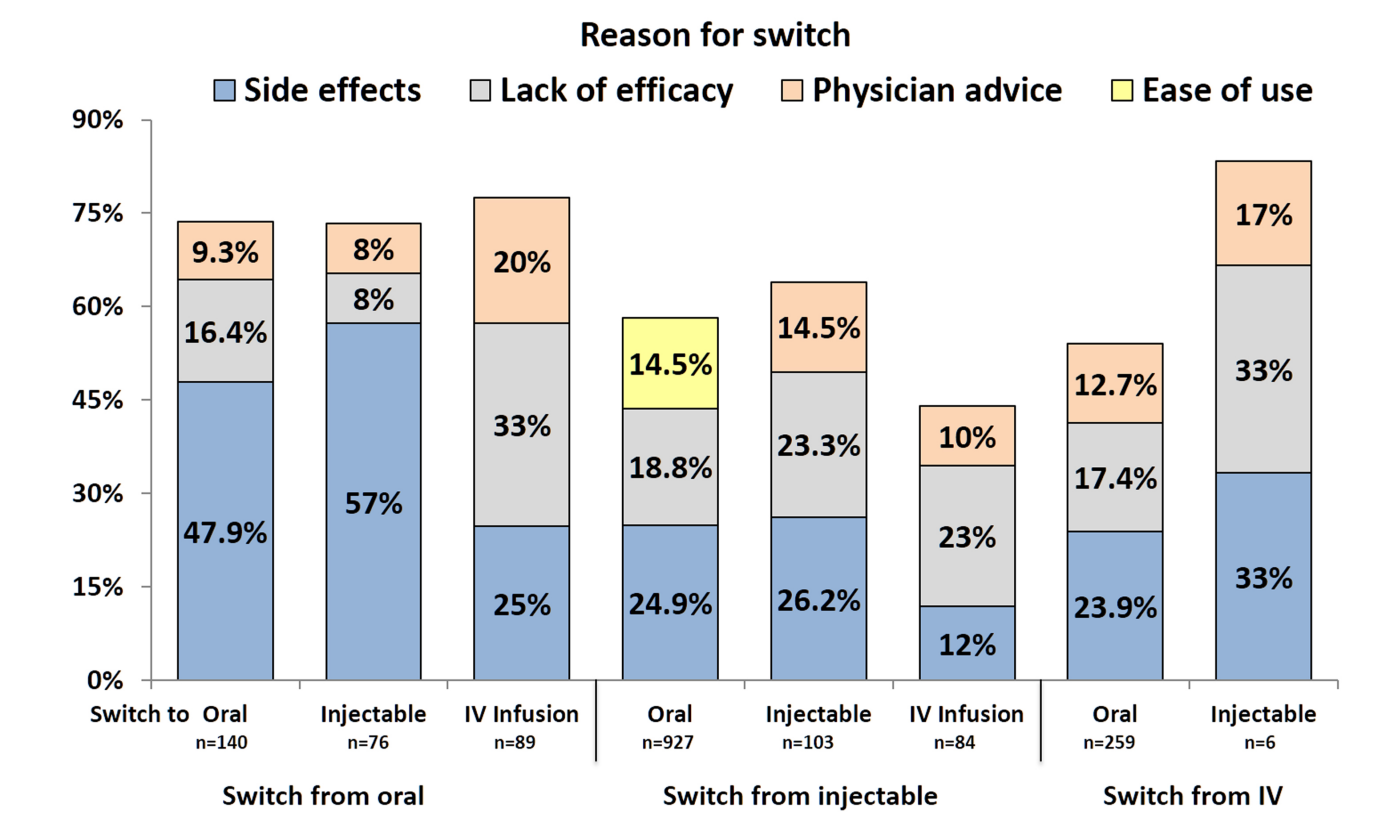
Three most frequent reasons for switching according to starting therapy, expressed as percentages of the total number of each kind of switch. Percentages do not sum up to 100% because only the top three switches in each group are displayed.

By monitoring conversations, it was possible to identify a number of reasons for drug switching discussed among patients. Four reasons accounted for more than 90% (1130/1234) of all switches: severe side effects, lack of efficacy, physicians’ advice, and greater ease of use (Table 3). The three most frequent reasons for switching in each of the therapeutic classes are shown in Figure 4. Overall, as well as in all individual drug classes, side effects and lack of efficacy were the two most frequent reasons to switch to a different medication. Side effects were the largest single reason for switches to oral or injectable therapies and search for greater efficacy was the most important factor driving switches to IV therapies. The third most frequent reason overall for switches was physicians’ advice (Table 3). However, in driving switches from injectable to oral therapies, greater ease of use was a more common reason (Figure 4). Physicians’ advice played a greater role in switches from oral to IV infusion therapies and from IV infusion to injectable therapies than in other switches. Unspecified safety concerns (without reported side effects) were reported as a reason to switch by 3.08% (38/1234) of patients. Costs of medication were given as reason for switching by less than 1% (7/1234) of patients.

**Table 3:** Frequency of reasons for switching away from MS treatments.

Reason	Frequency, n (%)
Severe side effects of previous drug	464 (37.60)
Lack of efficacy of previous drug	310 (25.12)
Physician’s advice	193 (15.64)
Ease of use of new drug	163 (13.21)
Worsening quality of life	39 (3.16)
Safety concerns	38 (3.08)
Insurance issues	13 (1.05)
High cost	7 (0.57)
Other	7 (0.57)

## Discussion

The results from this pilot study indicate that social intelligence can be applied to outcomes research with the power to tease out facts not available by other analyses, such as retrospective analyses of claims databases, commonly employed in outcomes research. Using a combination of automated listening and filtering and analysis of data by life sciences-qualified analysts and physicians, it was possible to obtain data on MS patients’ personal experiences of their treatments and to generate a map of the most common reasons for switching between therapies.

The study had three objectives. The first was to test the applicability of social intelligence in outcomes research. Use of the Internet for medical information varies with the condition: patients with MS have among the highest rates of use at 60% to 80% of (US) patients compared with 35% of (Canadian) patients with spinal cord injury [18,19]. In a survey of information-seeking behavior among patients with MS, the Internet (unspecified sites) was considered one of the most reliable sources of information, second only to doctors [2]. Government health agencies and media scored worse.

Although published data are likely to lag behind the rapid increase in Internet use fed by the mobile revolution, choosing MS as the condition for our analysis was expected to provide a large dataset to analyze which could be assumed to represent a varied population and reduce the potential for bias. The large number of data points related to MS included in our sample shows that there is an extensive and active social media population generating sufficiently large datasets with relevant information to enable analysis of questions typically posed in outcomes research.

The second objective of the study was to validate the representativeness of the social media population for MS. A comparison of the characteristics of the social media population with other MS patient cohorts characterized in community surveys or by analysis of large US administrative claims databases showed strong similarities between populations identified with different methods. Likewise, the analysis of switching patterns yielded similar results to those from other sources: market analyses by Novartis (data on file) indicate that most switches in real life are from injectable to oral therapies, including a not negligible percentage of patients on IV drugs. The similarities between these results from different sources indicate that the risk of bias in online data is no greater than that in acceptable and validated analyses of clinical reality in populations identified and characterized using other methods.

Most patients in the social media population had been living with their disease for several years and the age distribution corresponds to the means for MS patients on therapies. That a large majority of the population were women is a reflection of the demographics of the MS population because the disease is several times more prevalent in women [20]. Our rate of 76.73% women is similar to published proportions of female-to-male incidence rates in a number of populations including the United States [16], Sweden [21], and Canada [22]. There are molecular data in support of sex differences in responses to MS treatments [23]. Thus, the social intelligence population appears to be representative of those patients in real life who are mostly active in switching between therapies.

The third objective was to apply the social intelligence approach to the study of patterns of treatment switching between oral, injectable, and IV therapies for MS. For the purpose of the analysis, oral therapies were represented by the drugs Tecfidera, Gilenya, and Aubagio. In the time period covered by the data, these three compounds represented all available oral medications on the US market. Because the treatments represent different molecular entities, there is a reduced risk of potential bias from social media discussions of drug-specific characteristics not directly related to the oral or injectable nature of the different treatments. Injectable drugs were represented by five common BRACE therapies that together cover the majority of injectable MS medications in the United States. This pilot analysis did not attempt to tease out drug-specific reasons for switching, although the performance of the analysis indicates that such an exercise would be possible with the methods employed in this analysis.

There are few published data on patients’ reasons for switching between MS therapies. Most investigators have focused on efficacy comparisons in patients switching from one treatment to another, using observational data from registries to approach the real-world situation [24]. Our findings indicate that patients experience injectable therapies as associated with more side effects than oral medications or at least that patients were more willing to try oral drugs than to switch to other injectable or IV alternatives. The switches to IV drugs were mostly motivated by a desire for greater efficacy, with a small percentage of patients on oral medications apparently willing to risk a less favorable safety profile for greater efficacy with IV drugs. That physicians’ advice was a greater factor in switches involving IV therapies than in other switches is a reasonable finding, given the novelty and limited experience with these drugs [25]. However, because 80% of all switches were to oral therapies, the sample size for other switches may be too small to avoid selection bias or chance findings. There were very few switches to injectable drugs from other classes.

The strength of the method lies in the combination of automated and manual analysis. Manual analysis alone is too labor-intensive to be feasible in social media analysis of large datasets. Automated tools for content analysis will not capture the varieties of human expression, such as irony or the use of nonstandard abbreviations, nor will they identify complex semantic relationships between concepts or process information expressed in colloquial language typical of social media [26,27]. The blend of automated listening and human content analysis used in this research was designed to overcome these limitations and reduce the risk of misinterpretation.

The study has a number of limitations. First, all social media are susceptible to misinformation (eg, user experiences or unverifiable data sources), which may be difficult to identify even for the human analysts and guiding physicians [28]. There is a potential for bias in that certain types of patients may be more motivated to interact on social media than others, even if the huge size of the US social media population and the representativeness of our study population argue against this for this study. Because the data originate from public forums, medication-specific discussions on confidential websites, such as physician/patient discussion forums, have not been captured. A further limitation to analyses of social networks is the relative lack of socioeconomic and demographic information available [29]. There is a risk of duplication of data among our population because patients were identified by their screen names and the same patient may have used different screen names on different social media. It is also unclear how well social media data are representative of populations for other diseases than MS.

Although these limitations should be acknowledged, our analysis shows that when applied to appropriate questions that are frequently discussed openly by patients, social intelligence can be a powerful tool for outcomes research, providing information on specific factors driving patient’s health-seeking behavior that may not be obtainable by other means.

## Abbreviations

BRACE: Betaseron, Rebif, Avonex, Copaxone, Extavia
HIPAA: United States Health Insurance Portability and Accountability Act
IV: intravenous
MS: multiple sclerosis
STROBE: Strengthening the Reporting of Observational Studies in Epidemiology
